# Evaluation of Antidiabetic Activity of Hydroalcoholic Extract of *Cestrum nocturnum* Leaves in Streptozotocin-Induced Diabetic Rats

**DOI:** 10.1155/2013/150401

**Published:** 2013-09-16

**Authors:** Anil Kamboj, Sunil Kumar, Vipin Kumar

**Affiliations:** Institute of Pharmaceutical Sciences, Kurukshetra University, Kurukshetra 136119, India

## Abstract

*Objective*. To investigate antidiabetic activity of hydroalcoholic extract of *Cestrum nocturnum* leaves in Wistar rats. *Method*. *Cestrum nocturnum* leaves extract in hydroalcoholic solution were prepared by Soxhletation method and stored in refrigerator at 4°C for two days before use. Wistar rats were made diabetic by a single dose of streptozotocin (150 mg/kg i.p.). Hydroalcoholic leaves extract of *Cestrum nocturnum* was screened for antidiabetic activity and given to the STZ-induced diabetic rats at a concentration of 200 mg/kg and 400 mg/kg of body weight in different groups of 6 diabetic rats each orally once a day for 15 days. Metformin is also given to another group to support the result at a dose of 10 mg/kg of body weight orally once a day for 15 days. Blood glucose levels and body weights of rats were measured on 0, 5, 7, and 15th days. *Results*. Oral administration of the extracts for 15 days caused a significant (*P* < 0.01) reduction in blood glucose levels in diabetic rats. The body weight of diabetic animals was also improved after daily administration of extracts. The extract also improved other altered biochemical parameters associated with diabetes. Also the changes in food intake, water intake, and weight of internal organs were also restored to normal by the prolonged effect of extract treatment.

## 1. Introduction

Medicinal plants continue to provide valuable therapeutic agents, both in modern and in traditional medicine. As powders, extracts, decoctions, or infusions, plants are being used in the traditional systems of medicine in many parts of the world, especially in rural communities, for the control, management, and/or treatment of a variety of human and animal ailments. The current worldwide trends towards utilization of plant-derived natural remedies have, therefore, created a dire need for accurate and up-to-date information on the properties, uses, efficacy, safety, and quality of medicinal plant products [1]. The plant kingdom has become a target for the search by multinational drug and biologically active lead compounds [2].

Diabetes mellitus is a chronic metabolic disorder, mainly characterized by disruption in carbohydrates, protein, and fat metabolism caused by the complete or relative insufficiency of insulin action [3]. When the amount of blood glucose in the blood increases, for example, after a meal, it triggers the release of the hormone insulin from the pancreas. Insulin stimulates muscle and fat cells to remove glucose from the blood and stimulates the liver to metabolize glucose, causing the blood sugar level to decreases to the normal levels, as glucose is not metabolized; high amount of glucose is circulating in the blood (hyperglycemia). To keep the normal level of glucose in blood, the kidney removes the extra sugar from the blood and excretes it in the urine. Because glucose is not utilized by the body cells, the body is under constant impression of hunger, and that is why diabetics feel increased appetite (polyphagia) and eat more frequently [4].

Diabetes mellitus is one of the most common chronic diseases in the whole world. It is a complex, multifactorial disease which affects the quality, quantity, and style of an individual's life [5]. The fact confirmed by reports from the World Health Organization (WHO) shows that India has the largest number of diabetic subjects in the world [6]. Hyperglycemia can be handed initially with oral synthetic agent and insulin therapy. But these synthetic agents produce some serious side effects and are relatively expensive for developing countries [7]. The toxicity of oral antidiabetic agents differs widely in clinical manifestations, severity, and treatment [8]. In the natural system of medicine, many plants have been claimed to be useful for the treatment of diabetes mellitus. The dependence of large rural population on medicinal plants for treatment of diabetes is because of its availability and affordability [9]. Additionally, after the approbation made by WHO on diabetes mellitus, exploration on hyperglycemic agents from medicinal plants has become more significant [10].

So, the present study was conducted to evaluate antihyperglycemic activities of *Cestrum nocturnum *leaves in streptozotocin induced diabetic rats.

*Cestrum nocturnum*, family: Solanaceae, is an evergreen woody shrub growing to 4 metres (13 ft) tall. The leaves are simple, narrow lanceolate, 6–20 cm (2–8 in) long and 2–4.5 cm broad, smooth, and glossy, with an entire margin. The flowers are greenish white, with a slender tubular corolla 2–2.5 cm (1 in) long with five acute lobes, 10–13 mm diameter when open at night, and are produced in cymose inflorescences. A powerful, sweet perfume is released at night. The fruit is a berry 10 mm long by 5 mm diameter, the colour of an aubergine. There is also a variety with yellowish flowers. The plant contains many flavonoids and sterols/triterpenoids as its main constituents, which are known bioactive principles for antidiabetic potential [11, 12]. Flavonoids are also known to regenerate the damaged *β*-cells in diabetic mice [13, 14].

## 2. Materials and Methods

### 2.1. Collection of Plant Material

The aerial parts of plant were collected from Kurukshetra University campus, Kurukshetra, during November 2012 and identified as *Cestrum nocturnum* (family: Solanaceae) by Dr. H. B. Singh, Scientist Incharge, Raw Materials and Museum, National Institute of Science Communication and Information Resources, New Delhi, where a voucher specimen (no. NISCAIR/RHMD/Consult/-2011-12/2004/12) had deposited.

### 2.2. Preparation of Extracts

Aerial parts of *C. nocturnum* were dried in shade for two weeks. Dried parts were crushed and stored in an air-tight container at room temperature. Dried powder was then extracted with hydroalcoholic (70 : 30) solution using Soxhlet's apparatus.

### 2.3. Phytochemical Screening

The extract was subjected to phytochemical analysis to test the presence of carbohydrates, glycosides, alkaloids, flavonoids, tannins, sterols, and triterpenoids in leave extracts [15].

### 2.4. Animals

Wistar rats (200–250 gm) were selected for experimental study. The animals were kept and maintained under laboratory conditions of temperature (21.5 ± 22°C), humidity (60 ± 1%), and 12-hour light/dark cycle. They were allowed free access to food (standard pellets) and water *ad libitum*. Experimental protocols and procedures used in this study were approved by the Institutional Animal Ethics Committee of Kurukshetra University, Kurukshetra, India.

### 2.5. Induction of Diabetes

Hyperglycemia was induced by injecting streptozotocin at a dose of 150 mg/kg i.p. The animals were kept under observation. After 48 hrs, the animals were tested for glucosuria using Diastex strips [16]. Twelve days after the STZ injection, rats with fasting blood glucose levels greater than 200 mg/dL were considered diabetic.

### 2.6. Treatment Protocol

Diabetic animals were randomly assigned into the following groups of six animals each and treated as follows.

 Group I: normal control received 5 mL/kg of normal saline.
 Group II: diabetic control received vehicle (Tween 80, 5% v/v and 5 mL/kg of normal saline).
 Group III: diabetic rats received metformin (10 mg/kg).
 Group IV: diabetic rats received hydroalcoholic extract of *C. nocturnum* (200 mg/kg).
 Group V: diabetic rats received hydroalcoholic extract of *C. nocturnum* (400 mg/kg).

The drug solutions or vehicle were administered orally by gastric intubation once daily at 11 o'clock for 15 days. The effect of vehicle, extract, and standard drug on blood glucose and body weight was determined in animals.

### 2.7. Statistical Analysis

All values of results are presented as mean ± standard error of mean (SEM). The statistical analysis involving two groups was evaluated by means of Student's *t*-test, whereas one way analysis of variance (ANOVA) followed by Dunnett's multiple comparison posttest was used for statistical comparison between control and various treated groups. Statistical significance was accepted at the *P* < 0.05 values.

## 3. Result

Phytochemical analysis of *Cestrum nocturnum* leaves showed the presence of flavonoids, tannins, saponins, sterols, and triterpenoids. Flavonoids are also known to regenerate the damaged *β*-cells in diabetic mice [14, 17]. The effects of metformin and hydrochloric extracts on blood glucose levels in normal and diabetic rats after treatment of 15 days are shown in Table 1, in which all extracts showed significant reduction (*P* < 0.01). It was observed that standard drug metformin lowered the blood glucose levels significantly bringing it back to normal which is an indication of the presence of some *β*-cells, as metformin is known to stimulate insulin secretion from *β*-cells. A significant reduction in average weight was observed in STZ-induced diabetic rats (Table 1). The decrease in weight in diabetes was due to continuous excretion of glucose and decrease in peripheral uptake of glucose and glycogen synthesis [18]. Increase in body weight and decrease in blood glucose might be due to improving the glycemic control mechanisms and insulin secretions from remnant pancreatic cells in diabetic animals.

## 4. Discussion

In the present study, *Cestrum nocturnum* was selected for antidiabetic studies owing to its traditional uses. Therefore, the study was undertaken to justify its claimed uses. Wistar rats were selected as experimental animals for the antidiabetic activity. The extract was screened for streptozotocin-induced antidiabetic activity. The hydroalcoholic extract of plant showed significant (*P* < 0.01) antidiabetic activity at both doses, that is, 200 and 400 mg/kg of body weight. This is further evidenced by percentage reduction in blood glucose levels after 15th day after administering the extract at both of the doses. The hydroalcoholic extract significantly increased the body weight of diabetic animal at higher doses.

During this prolonged study, various physical parameters were also observed such as body weight, food intake, water intake, and weight of internal organs. Generally, body weights are reduced in diabetic animals, but in this study, the decrease in body weights was diminished by the extract treatment; thus this effect may be useful for the diabetic animals. The phytochemical study showed the presence of saponin glycosides, steroids, and phenolic compounds in the extracts, which might be a reason for the good activity of extract.

*Cestrum nocturnum* hydroalcoholic extract showed antidiabetic effect comparable to various plants extracts like *Dillenia indica* [7, 8], *Callistemon lanceolatus* [19], and *Cassia siamea* [20] as reported in our previous research work. However, this is a preliminary work, and more work is needed to determine the active ingredients in the extract which may help in improving management of the antidiabetic agents. The study reveals that the hydroalcoholic extract of *C. nocturnum* could be added in list of herbal preparation, beneficial in diabetes mellitus. *Cestrum nocturnum *can be considered as an important addition to the therapeutic armamentarium for the treatment of diabetes. Further studies can be undertaken at the cellular and molecular levels, which may further elucidate its mechanism in detail. The present investigation has also opened avenue for further research especially with reference to the development of potent formulation for diabetes mellitus from *Cestrum nocturnum.*

## Figures and Tables

**Table 1:** Effect of *Cestrum nocturnum* on blood glucose and body weight.

Group	Blood glucose (mg/dL)				Body weight (g)			
	Day 0	Day 5	Day 10	Day 15	Day 0	Day 5	Day 10	Day 15
Group I	108.1 ± 2.1	137.3 ± 1.9	148.4 ± 3.2	112.1 ± 1.3	210.6 ± 1.2	212.17 ± 0.8	218.2 ± 0.6	208.4 ± 0.9
Group II	287.1 ± 1.6	295.3 ± 2.9	312.6 ± 6.1	322.3 ± 1.2	216.1 ± 1.5	209.12 ± 1.1	201.2 ± 1.1	190.6 ± 0.02
Group III	279.1 ± 12.9	236.1 ± 8.1*	181.5 ± 1.7*	145.2 ± 3.9	226.9 ± 0.8	206.2 ± 1.2*	201.3 ± 0.9	198.9 ± 1.7
Group IV	281.5 ± 20.5	215.8 ± 3.1*	164 ± 3.2*	117 ± 1.6	222.3 ± 0.6	213.3 ± 1.0**	201 ± 1.4	193.6 ± 0.8**
Group V	284.2 ± 7.1	235.5 ± 1.4**	115.02 ± 1.1**	98.02 ± 3.1	228.4 ± 0.7	216.1 ± 0.1**	211.2 ± 2.6	206.2 ± 1.5**

Data represent mean ± SEM. *P < 0.05 and **P < 0.01 when compared with group II.
